# Modeling Users, Context and Devices for Ambient Assisted Living Environments

**DOI:** 10.3390/s140305354

**Published:** 2014-03-17

**Authors:** Eduardo Castillejo, Aitor Almeida, Diego López-de-Ipiña, Liming Chen

**Affiliations:** 1 Deusto Institute of Technology—DeustoTech, University of Deusto, Avda. Universidades 24, Bilbao 48007, Spain; E-Mails: aitor.almeida@deusto.es (A.A.); dipina@deusto.es (D.L.-I.); 2 School of Computer Science and Informatics, Faculty of Technology, De Montfort University, Gateway House, The Gateway, Leicester, LE1 9BH, UK; E-Mail: liming.chen@dmu.ac.uk

**Keywords:** AAL, human-computer interaction, user, context, device, modeling, context-awareness, user capabilities

## Abstract

The participation of users within AAL environments is increasing thanks to the capabilities of the current wearable devices. Furthermore, the significance of considering user's preferences, context conditions and device's capabilities help smart environments to personalize services and resources for them. Being aware of different characteristics of the entities participating in these situations is vital for reaching the main goals of the corresponding systems efficiently. To collect different information from these entities, it is necessary to design several formal models which help designers to organize and give some meaning to the gathered data. In this paper, we analyze several literature solutions for modeling users, context and devices considering different approaches in the Ambient Assisted Living domain. Besides, we remark different ongoing standardization works in this area. We also discuss the used techniques, modeled characteristics and the advantages and drawbacks of each approach to finally draw several conclusions about the reviewed works.

## 1. Introduction

Personalization and recommender systems have spread thanks to the growth of heterogeneous connected devices and their increasing possibilities. Daily we carry with us different smart objects whose capabilities allow them to be aware of the environment. These devices can actually learn from experience. They are aware of our behavior, preferences and trends. This new and rich situation enhances and improves the way users interact with context, but it also makes people more dependent in the use of these technologies. This also means that users are becoming a significant and active part of the Ambient Assisted Living (AAL) and Pervasive Computing environments. For example, we can obtain recommendations while shopping based on the previously purchased items, change the music based on the user's mood, get notifications of new and interesting releases, receive different alternatives to reach some places considering our current location, even get social advices about people consuming the same or similar products or services. Besides, this is not a situation which concerns only researchers. Local governments and public administrations have discovered the importance of working with context data [1]. This way, they try to improve citizens' satisfaction through new and smarter infrastructures.

On the other hand, application and service personalization has abandoned the idea of being just tools or frameworks to allow users to customize different resources and devices. Actually it involves the perspective of inclusive design, whose main purpose is to avoid side lining smaller groups of users, like people with disabilities and elders [2]. These groups are known for suffering from several limitations. Moreover, although nowadays the share of people aged 65 represent a 17% of the current European population, by the year 2060 this figure is projected to rise to 30% [3]. As a consequence, and as the European Commission states, “*the EU would move from having four people of working-age to each person aged over 65 years to about two people of working-age*”. Evidently the current situation shows that it is still a small group, but with a high expected increasing ratio. Nevertheless, this implies that we are still in time of accommodating, adapting and overtaking for future economic and demographic consequences.

Then, what is the right way of considering context, users and devices to perform these recommendations and adaptations for the user? The answer is *modeling*. Modeling these entities allow researchers and developers to consider different conditions that might trigger several recommendations, adaptations or services to satisfy the user needs. User's interests are useful for recommending systems [4]. On the other hand, their medical capabilities might be needed for adaptive environments [5]. The same occurs when we consider context or devices.

As we will see later in Section 2, during the past 15 years there has been a lot of work done in this area. Authors have followed different approaches and developed different techniques to take into account every possible scenario. In this paper, we analyze these solutions studying their advantages and disadvantages to, finally, discuss about the evolution of these systems and about the future of modeling.

The remainder of this paper is structured as follows: First, in Section 1.1 we introduce our motivation for this paper to detail the perspective from which this work should be considered. In Section 2 we review the literature solutions for modeling context, users and devices during the past 15 years. Within each subsection of the state of the art an analysis of the considered literature solutions is presented. Several standardization works are also remarked. Next, Section 3 discusses about the presented approaches and remarks several problems and future issues to be taken into account. Finally, in Section 4, we summarize our experiences and discuss the conclusions.

### 1.1. Motivation: Human-Computer Interaction and Users’ Context Disabilities

**Definition 1.** *Designing an object to be simple and clear takes at least twice as long as the usual way. It requires concentration at the outset on how a clear and simple system would work, followed by the steps required to make it come out that way—steps which are often much harder and more complex than the ordinary ones. It also requires relentless pursuit of that simplicity even when obstacles appear which would seem to stand in the way of that simplicity [6]*.

This cite by Ted Nelson [6] in 1977 already pointed out the problems that designing a product entails. One of the most significant issues to face during this process is the usability. According to the ISO/IEC 9126 standard, quality represents a property of the software product defined in terms of a set of interdependent attributes, i.e., usability, security, reliability, performance, complexity, readability and re-usability expressed at different levels of detail and also taken into account the particular context of software use [7,8]. At this point, the ISO 9241-11 standard [9] states that usability is the extent to which a product can be used by specified users to achieve specified goals with effectiveness, efficiency and satisfaction in a specified context of use.

**Figure 1.**
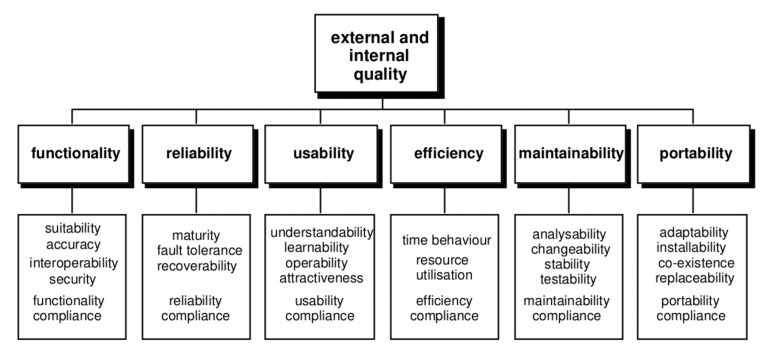
Quality model for external and internal quality [7].

**Figure 2.**
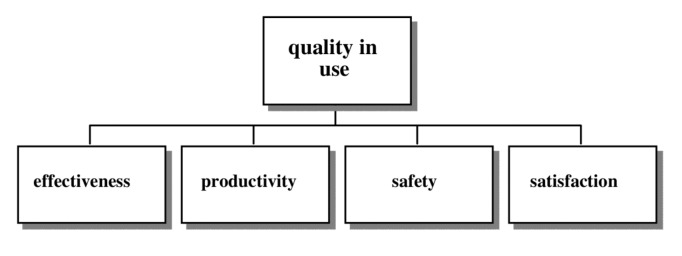
Quality model for quality in use [7].

The interaction with devices needs to be satisfactory for the users. The ISO/IEC 9126-1 [7] presents and details a two-part model for software product quality:

1. Internal and external quality (see Figure 1): Internal Quality is the totality of attributes of the software product from an internal view, e.g., spent resource. It is measured and improved during the code implementation, reviewing and testing. External Quality is the quality when software is running in terms of its behavior, e.g., number of wrong expected reactions. It is measured and evaluated for software testing in a simulated environment [8].
2. Quality in use (see Figure 2): It is the capability of the software product to enable specified users to achieve specified goals with effectiveness, productivity, safety and satisfaction in specified contexts of use.

However, the design process becomes troublesome because of the nature of each user. Users are very different from each other. They like different things and they sense and perceive in different ways. Moreover, they have different capabilities. Besides, there are several groups which suffer these differences more deeply: people with disabilities and the elderly. The first group suffers from different disabilities which are responsible for limiting several capabilities in a certain way. For example, users with sight disabilities will suffer from interaction problems with their devices if this interaction is based on visual stimulus, e.g., using a device display. On the other hand, elderly people usually suffer from similar interaction troubles due to their aging. As their senses tend to tire their capabilities and interaction levels decrease [5]. Current technology trends try to reduce the interaction barriers that elderly suffer with current devices. Mobile phones have audio control interaction and screen magnification, TVs have zoom and subtitles capabilities, *etc*. Nevertheless, the elderly are used to the products they already know [10,11]. For these groups the designed devices should be:

- *Easy to use*, so the users are able to use them to their own purposes.
- “*Easy to learn*”. This way, the final purpose of the device should be affordable in an acceptable time interval.
- *Easy to recall*, so the users are able to remember how to interact with the device.

Nonetheless, we are not exempt of suffering from similar situations. There are many conditions which cause people without disabilities to feel like they have one. Making a phone call in a very sunny day or send an email when it is raining are examples of several context situations which limit users' capabilities. These circumstances also introduce what context might be capable of during an interaction process. Desktop environments are known for being less prone to suffer from context conditions (obviously certain situations are impossible to avoid, like infrastructure problems). On the other hand, mobile devices are predisposed to experience problems due to current context situation.

## 2. Analysis of the State of the Art

In this section, the most significant user, context and device models and solutions for the past 15 years are analyzed. First, Section 2.1 focuses its attention in the models that have been used for characterizing users and their capabilities. Section 2.2 analyses the most significant context-aware systems and the way these approaches model the context and its characteristics. Devices are also one of the main entities that are taken into account in this paper as their capabilities and characteristics are vital for performing any interaction process. Therefore, Section 2.3 examines several modeling techniques and the devices' capabilities in different domains. Besides, a chronological review of the evolution of the presented models will be depicted in each section.

### 2.1. Users

#### 2.1.1. A Chronological Review of the Evolution of User Models

The following figure illustrates the evolution and different solutions by chronological order for the last 15 years. During these years different user characteristics have been taken into account considering the final purpose of the designed system.


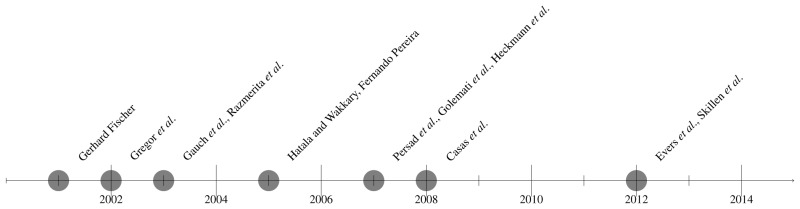


First user modeling systems started in the late eighties. Allen [12], Cohen and Perrault [13,14] and Elaine Rich [15,16] are examples of researchers whose works inspired next user modeling approaches. From there several authors started collecting different types of information about the users and exhibiting, for example, different kinds of adaptations to them [17].

However, first, what is user modeling? There are at least two different perspectives which might answer this question. One of these is related to the Artificial Intelligence research area, and it considers user modeling as the process through which systems gather information and knowledge about users and their individual characteristics. Therefore, a user model is considered a source of information about the user of the system which contains several assumptions about several relevant behavior or adaptation data. However, in this paper the Human-Computer Interaction research perspective is taken, which is defined by Wolfgang Pohl [18] as follows:

**Definition 2.** *It refers to an a-priori model of the users of a computer system that the system designer has in mind, or to the assumed models that users will probably develop of the system and the tasks they can perform using the system*.

Nevertheless, this definition and the AI perspective coincide in the idea that every system must use information about the user to be able to see and react to their different problems and needs and improve the system purpose. This section analyses the most significant user models in the past 15 years.

##### 2001, Gerhard Fischer

In 2001, Gerhard Fischer [19] reviews the user models of the past 10 years. He describes how using computers in Human-Computer Interaction (HCI) environments has been always modeled as a user-computer couple. These elements are modeled as an explicit connection which represents the communication between them. New and modern interfaces such as windows, menus, pointers, colors, sound and touch screens have “enlarged” this communication line thanks to their capabilities. Furthermore, in addition to the possibilities of new design approaches, knowledge-based architectures in HCI explore the possibility of implicit communication channel. The required knowledge considers the problem domain, communication processes and the communication agent. Users are part of the communication agent group. Fischer defends the idea that there are many types of users. Besides, their needs change with the experience and through time. This way, a simple user classifications, e.g., “novel”, “intermediate” and “expert”, is not enough to characterize users in complex environments. Nevertheless, despite Fischer remarks the significance of each agent, he does not establish which agent capabilities are important to face the problem of modeling a user.

##### 2002, Gregor *et al.*

In 2002, Gregor *et al.* [5] focus their approach on a certain group of users: the elderly. A three group classification is presented. In the first group there are the fit older people, who are not considered disabled. The second group is formed by older fragile people who have one or more disabilities. Finally, the last group encompasses the older and people with disabilities whose capabilities to function depend on other faculties. In this case, the authors identify several user capabilities:

- Physical, sensory and cognitive capabilities.
- The ability to learn new techniques (cognitive).
- Memory problems (cognitive).
- The environment can affect several elderly capabilities.
- Elderly experience (as a positive fact).

On the other hand, Gregor considers that, as people grow older, their capabilities change. This process encompasses a reduction of cognitive, physical and sensory functions depending on the individual. This “dynamic diversity” is a significant issue for modeling users and designing computing systems.

##### 2003, Gauch *et al.*

Towards the goal of personalized navigation of online information Gauch *et al.* [20] provide a user ontology for dynamically modeling the user browsing. The ontology is formed by several concepts which are weighted indicating the user's perceived interest in the corresponding concept. These concepts are related with surfing experience, *i.e.*, the content, length and time spent, on each Web page and classified into the reference ontology. This way the user profile is created automatically. This means that the user profile information is collected implicitly without user feedback, as the ontology's concepts are automatically weighted considering the amount of related information from the user browsing.

##### 2003, Razmerita *et al.*: The OntobUM Ontology

Focused in the context of Knowledge Management Systems (KMS) Razmerita *et al.* [21] present OntobUM, a generic ontology-based architecture for user modeling. The model is generated through two different ways:

- Explicitly, using a user profile editor. This way the user has to provide some information.
- Implicitly, information maintained by several intelligent services which (1) maintain and update the information about the user considering the user's behavior with the services and (2) provide adapted services based on user's preferences.

The architecture of the presented ontology is composed of the following ones:

- The User Ontology, which structures the different characteristics and preferences of the user.
- The Domain Ontology, which defines several concepts about the domain.
- The Log Ontology, which manages the semantics of the interaction between the user and the whole system.

Authors identify several users' characteristics that are relevant for a KMS under the Behavior concept. Nevertheless, most of the user ontology is generic and it is available to be used in other application domains.

##### 2005, Hatala and Wakkary: The Ec(h)o System

Ec(h)o is an ontology-based augmented audio reality system for museums which aims to maintain rich and adaptive output information. The main purpose of this work is to address the problem of supporting experience design and functionality related to museum visits through user models combined with augmented reality and tangible user interface system. Hatala and Wakkary [22] find several challenges for capturing rich information about the context. For the presented museum scenario, social, cultural, historical and psychological factors are significant for the user experience. In this field the argumentation made by Dourish [23,24] is remarked as relevant. Dourish states that activities and context are directly and dynamically linked. This concept is called “embodied interaction”.

The core of the ec(h)o's reasoning module is a dynamically updated user model [25]. The ruled-based model changes as the user moves through the museum and selects several audio objects. This models enables developers to consider which inputs influence user interests. In the ec(h)o system there are two ways of updating the model: the user movement and a selection of an audio object. These actions have different effects on the model of the user interests, *i.e*, influence of initial interest selection, of object selection on user interest and of location change.

As it occurs with recommender systems, user's interest are vital for the concept ontology. These concepts are weighted in the ontology as concepts which represent the user's likes within the environment. Besides, an interaction history is maintained recording the way the user interacts with the museum. In addition to these characteristics the user type is also considered. This way the system is allowed to characterize the user experience with the environment. It classifies users into three different categories:

- The avaricious visitor, who wants to see as much as possible in a sequentially way.
- The selective visitor, who is more selective with the concepts he/she is interested in.
- The busy visitor, who prefers to not spend much time and get a general vision of the exhibition.

##### 2005, Fernando Pereira

Within a video adaptation and quality of experience evaluation scenario, Fernando Pereira [26] studies a user characterization through three different dimensions: sensorial, perceptual and emotional. First of all, Pereira establishes the difference between sensations and perceptions as follows:

- Sensations are monomodal, more low-level, physical and less related to the real world composition than perceptions. They regard the simple conscious experience for the corresponding physical stimulus, e.g., light variation and eyes reaction to this change. They are related to the first contact between a human and the surrounding environment.
- Perceptions are multimodal, and they are part of the cognition process (knowing and learning) and regard the conscious experience and identification of objects.

On the other hand, emotions are considered as central in a communication and entertainment process. Therefore, Pereira proposes a triple layered sensation-perception-emotion (SPE) user model for the evaluation of the quality of experience in the consumption of multimedia content.

##### 2007, Persad *et al.*

Persad *et al.* [27,28] relate user capabilities and product demands as a tool to evaluate the product design. Inclusive design is defined as follows [2]:

**Definition 3.** *The design of mainstream products and/or services that are accessible to, and usable by, people with the widest range of abilities within the widest range of situations without the need for special adaptation or design*.

In a particular toaster case study, the sensory, cognitive and motor product demands are compared with different users capability levels. Authors remark four main components to consider when it comes to interaction between people and technology: (1) the user; (2) the product; (3) the environment or context and (4) the set of activities or tasks that define the interaction [28]. They try to assess an adaptation degree between users and the designed products using different compatibility measures. These measures can be assessed on different levels of human capabilities, including sensory, cognitive and motor. The concepts of user capability and product demand provide a useful framework for analyzing the user-device compatibility. The product demand levels are considered multidimensional and they are set by the interface attributes of the product itself. For example, a product's text display will be designed with a certain text size, font, and color contrast. The combination of these attributes define the visual demand level within the user visual capabilities. Similarly, other combinations of product attributes command several cognitive and motor demands. Authors also reviewed functional classifications and experimental studies to identify the most relevant low-level skills for designing products within the cognitive, motor and sensory domain [28].

##### 2007, Golemati *et al.*

Golemati *et al.* [29] present an ontology which considering past literature solutions aims to reduce the intrinsic problems of user modeling: ad-hoc modeling processes, the required amount of work to model users and the possibility of errors by omitting several user's characteristics. To this end, the authors present an extensible, comprehensive and general ontology which design is addressed through a top-down approach by firstly collecting static information about the user. Next, the ontology designers analyses the semantics of the profile models and suggest concepts that would adequately model them. It is remarkable that this work is focused on static user characteristics, although they consider the possibility to incorporate dynamic and temporal characteristics.

##### 2007, Heckmann *et al.*: The GUMO Ontology

A different approach is implemented by Heckmann *et al.* [30]. Divided into four main groups (emotional state, personality, characteristics and physiological state), the authors present GUMO, an ontology model to characterize users capabilities within adaptive environments. A significant user aspect that is taken into account in this work is the stress. In the adaptive interfaces domain it is needed to pay special attention to the consequences of each adaptation. But the stress is not only determined by this process. It is also derived from several user experiences, as the current context state, e.g., traffic, noise, surrounding people, *etc.* [31].

##### 2008, Casas *et al.*

Another approach is the one presented by Casas *et al.* [32]. In this case the authors work under the “Persona” concept which is introduced to distinguish between different user groups within an adaptive user interfaces domain. Originally this concept was introduced by Alan Cooper [33] in 1999 by the following definition:

**Definition 4.** *”Personas” are not real people, but they represent them through a design process. They are hypothetical archetypes of real users*.

Casas *et al.* distinguish between two categories of people:

- Primary: those who represent the main group and use primary interfaces.
- Secondary: those who can use primary interfaces, but with several extra needs.

By assigning random values to several characteristics, e.g., age, education, profession, family conditions, disabilities and technological experience, authors are capable of covering a wide range of potential users. However, the most significant contribution is that, instead of being focused on users capabilities, they consider on the users needs. To that end they build a user profile supported by four main bases:

- The user level, which indicates the ability of the user to face the system.
- Interface, for the interaction mechanism to be used by the user.
- Audio, to indicate the audio volume levels.
- Display, which includes usual display controls (contrast, colors, brightness…).

This approach is focused on the solution, on the adaptation itself. It is a perspective which allows users to configure the interaction based on their capabilities. This helps applications designers because user capabilities are not directly taken into account in the model as medical or technical aspects. This way there is no need to be experts or have any medical knowledge about users disabilities. Another advantage is that each user can manage his/her own profile. This way, they can configure their preferences and capabilities on their own.

##### 2012, Evers *et al.*

Several studies, as the research presented by Evers *et al.* [34], recognize that it is complex to perform interfaces adaptations without bothering the user. On the one hand, adapting an interface without the participation of the user might lead to an unsatisfactory result. On the other hand, asking too much for participation might bother the user. From this work we assume that if the user has high stress levels the corresponding application should not ask for interaction. This way, the application should operate as “automatic” and “self-sufficient” as possible. Following this stress perspective Liao *et al.* [35] present an unified probabilistic decision model based on Influence Diagrams for modeling user stress levels. These levels were inferred by probabilistic inferences of several sources data, e.g., heart rate, mouth openness, head movements, pupils monitoring, *etc*.

##### 2012, Skillen *et al.*

Within an application personalization within mobile environments Skillen et al. [36] present a User Profile Ontology which is able of modeling dynamic components. The ontology considers both static and dynamic aspects of the user mainly focused on his/her behavior changes. In this work user capabilities are also taken into account for the user profile. Capabilities are defined as the extent to which the user has an ability, *i.e.*, physical, emotional or cognitive, to carry out some activities. User's interests and several context parameters are also considered in the ontology to cover context-aware environments.

#### 2.1.2. Users Models Comparison

In this section, we emphasize our user modeling perspective. There are several points of view regarding the user modeling requirements. Here the HCI perspective is taken. This means, (as Pohl states in [18]) that the user model refers to user characteristics using a certain system (see Section 2.2.1.). In the AI perspective the works by Alfred Kobsa and Wolfgang Pohl are significant. The authors design generic user model systems (known as shell systems) under this viewpoint [17,37].

Now that the HCI viewpoint has been remarked, we emphasize the amount of different domains that are addressed in the literature considering user modeling. From product design to multimedia and user interfaces adaptation, every approach follows the same purpose: to consider several user characteristics to improve the system and user's satisfaction and product or service usability. However, although these solutions share the same objectives, the considered characteristics differ a lot. For ubiquitous and more context-aware domains, activities are taken into account. For example, Razmerita [21] *et al.* discuss about an ontology based architecture, which aims to be generic through collecting user data by two different ways (explicitly and implicitly). Golemati [29] *et al.* also take an ontological point of view to avoid the problem of domain dependency (among others) by designing a more general, comprehensive and extensible ontology.

Gauch *et al.* [20] remark in the presented ontology the importance of time. Regarding the studied domain (web browsing) time is significant because it might help characterizing the user considering the spent time reading an article or visiting a website. Well known and popular recommendation systems, as Youtube, utilize this information combined with different explicit and implicit sources from the user interaction to make proper recommendations [38].

User activities have also been considered as relevant for many authors in the literature. The first example is the Doppelgänger system [39] (1991), which uses activities for sharing relevant user information to different applications. In the same way, Persad *et al.* [27], Heckmann *et al.* [30] and Skillen *et al.* [36] modelled activities to take user's behavior and interaction into account for the proposed classifications and systems. For Hatala and Wakkary [22] activities are also vital within context-aware environments.

As occurs with context modeling (see Section 2.2.2.) many different techniques are available for the model representation. This usually depends either on the developer, because of his/her experience, or in the system's technical characteristics. For example, if the system is able to performs inference with the user data an ontology based representation could be more helpful than an object based one. Strang and Linnhoff-Popien [40] demonstrate that ontological modelling is more appropriate for ubiquitous computing environments.

It is also common to model medical related characteristics of the user. For example, the works by Gregor *et al.* [5] and Persad *et al.* [27] consider physical, cognitive an sensory capabilities. Skillen *et al.* [36] also model several user abilities for performing different tasks and activities. The problem is that being aware of these capabilities is difficult and, in some cases, poorly practical. For example, measuring the sight capability of one individual requires medical experience or advise. Besides, people with the same affection do not respond in the same way. A person who was born blind would interact differently with the environment than another who has been losing sight during his/her life. The precise same disability, e.g., tunnel vision, might affect different people in many different ways depending on their personal skills, e.g., adaptability, orientation, etc. This leaves an open issue which leads us to an idea. What if, instead of modeling medical skills (disabilities), we were able to model user's capabilities? The first approximation for this is found in Casas *et al.* [32] work. They present a user profile which abstracts from physiological aspects and let users to manage and configure their own profile. Just in the opposite, Skillen *et al.* [36] model user capabilities as a set of abilities which allow users to perform some task or activities.

On the other hand, Gerard Fischer comments in [19] that it not only is difficult to model users because of the wide range of different types of people that exist. He also considers that each individual changes with experience and through time. For example, old people's capabilities decrease with ageing. This idea is shared with Gregor *et al.* [5], whose work is centred around the elderly. Heckmann [41] not only considers that users might evolve (from an AI perspective), but he also takes new context information from the inference process. This also opens a new point of view that we address in Section 1.1 and it takes context as a significant user's environment entity that might directly influence the user's capabilities. In other words, users change through experience, time and, in concrete situations, due to the current context characteristics. For example, an individual might not suffer from any mobility disability, but in a crowded street would be difficult to perform several daily activities (just walking could be difficult). Razmerita [21] *et al.* also address this issue when they talk about the implicit user information collecting process. This, of course, deals with the concept of dynamism.

Evers *et al.* [34] consider that respecting user's interactive behavior with applications needs to be taken into account. On the other hand, Pereira [26] analyses the differences between emotional and perceptional user characteristics.

Table 1 summarizes the analyzed approaches for user modeling emphasizing the modeled user characteristics and domains. Besides, although in a first version every used technique was remarked, for this paper we have focused on emphasizing just those which follow an ontology-based approach. This is because many of the cited works are more theoretical or surveys, or they just give some advices about important context data when facing a context modeling task. Besides, Strang and Linnhoff-Popien [40] demonstrate that using ontologies is more appropriate for modeling context-aware systems.

### 2.2. Context

Context is mostly defined by the definition by Anind K. Dey [42] as follows:

**Definition 5.** *Context is any information that can be used to characterize the situation of an entity. An entity is a person, place, or object that is considered relevant to the interaction between a user and an application, including the user and applications themselves*.

Their definition enables developers to easily enumerate those elements which take part in the context for a certain application domain. The author also defines that *“if a piece of information can be used to characterize the situation of a participant in an interaction, then that information is context”*. By a proposed example it is seen how different pieces of information are analyzed to determine if they belong to what Dey states context is. These definitions are based on research experience. This section shows how modeling and defining context has evolved in the past 15 years.

Context management allows us to identify the conditions of the environment. This way, developers are able to adapt services or applications for the user taking into account these conditions. To do this first there is the need of gathering context information. Next, this information has to be somehow processed and, finally, it will be used to personalize and contextualize the current situation.

#### 2.2.1. A Chronological Review of the Evolution of Context Management

The following figure shows the evolution for context modeling by chronological order for the last 15 years.


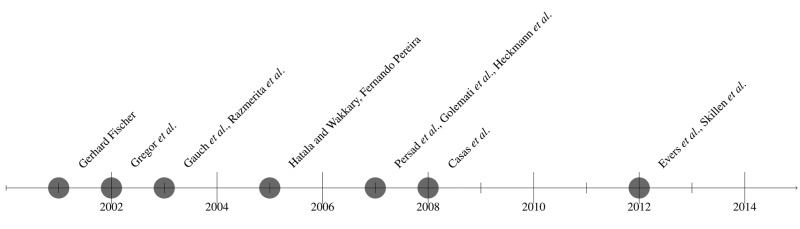


Dey [42] also defines *context-aware systems* as those systems which, using context data, provide significant information and/or services to the user where the relevancy of the given information depends on the user task. This domain dependency implies that each solution or context model is too concrete or specific and poorly extensible to other environments. Schmidt *et al.* [43] consider several issues about context modeling emphasizing the excess of abstraction about context-aware systems and environments which causes a lack of models to be compared. Therefore, a categorized model for context-aware systems is presented. In 2001 Anthony Jameson [44] studies how context-aware computing represents a challenging frontier for researchers. In this work information about the environment, the user's current state, longer term user properties and the user behavior are compared in order to take the correct adaptation decision. Several research investigations focused on user interface adaptation area base their processes on context changes as triggers. However, they lack of a common model of context in their platforms [45,46]. This fact emphasizes the argument established by Schmidt *et al.* [43].

In this section, a review of the most popular context-aware systems is presented, mainly focused on the modeled context parameters, techniques, domains and dependencies. Besides, several authors define context and context-awareness through their own experience.

##### 2000, Chen and Kotz

Chen and Kotz [47] define context as follows: *Context is the set of environmental states and settings that either determines an application's behavior or in which an application event occurs and is interesting to the user*. Besides, an active and passive definitions of context-aware computing are also given:

- Active context awareness: *“an application automatically adapts to discovered context, by the changing in the application's behavior”*.
- Passive context awareness: *“an application presents the new or updated context to an interested user or makes the context persistent for the user to retrieve later”*.

Based on the work by Schilit *et al.* [48], Chen and Kotz consider time as an important and natural context feature for many applications. Besides, they introduce the term *“context history”*, which is an extension of the time feature recorded across a time span.

A significant problem remarked in this work is the impossibility to exchange context information between the studied context-aware systems due to the way they model the environment. Furthermore, as location is one of the most modeled context features, Chen and Kotz provide a study of several aspects that should be taken into account when researchers face the problem of modeling it.

##### 2001, Anthony Jameson

Anthony Jameson [44] analyses in 2001 how context-aware computing represents a challenging frontier for researchers in the field of Adaptive User Interfaces. In this work information about the environment, the user's current state, longer term user properties and the user behavior are compared in order to take the correct adaptation decision. The idea is to compare several scenarios. In the first one, only information about the environment is considered. In the following scenarios the user's current state, behavior and long-term properties are taken into account. This way, the results conclude that considering a wider range of user information can help context-aware systems designers.

##### 2002, Henricksen *et al.*

The approach followed by Henricksen *et al.* [49] makes the following notes about context in pervasive environments:

- Context information exhibits temporal characteristics. Context can be static (those characteristics that do not change over time, e.g., birthday) or dynamic, e.g., user location. Besides, the persistence of the dynamic information can easily change. This way, it is justified that the static context should be provided by the user, while the dynamic one should be gathered by sensors. Past historic and a possible future context are also taken into account as part of the description of the whole context description.
- A second property of the context information is its imperfection. Information can be useless if it cannot reflect a real world state. It also can be inconsistent if it contains contradictions, or incomplete if some context aspect are unknown. There are many causes to these situations. For example, information can change so fast that it may be invalid once it is collected. This is obviously because the dynamic nature of the environment. Besides, there is a strong dependency on software and hardware infrastructures, which can fail any time.
- Context has multiple alternative representations. Context information usually comes from sensors which “speak” different languages. For example, a location sensor can use latitude and longitude metrics while the involved application works with a street map.
- Context information is highly disassociated. There are obvious connections between some context aspects, e.g., users and devices. However, other connections need to be computed with the available information.

This work also indicates the dependency between context models, the scenarios and use cases of the application domains. Authors extract several context parameters to consider:

- User activity, distinguishing between the current one and the planned one.
- Device that is being used by the user.
- Available devices and resources.
- Current relationships between people.
- Available communication channels.

##### 2002, Held *et al.*

In 2002, Held *et al.* [50] discuss about the necessity of content adaptation and representation formats for context information in dynamic environments. A significant perspective is the justification of modeling not only the user but the network status and device context information as well. The following parameters are considered as relevant context information:

- Device: basic hardware features, e.g., CPU power, memory, …, user interface input, e.g., keyboard, voice recognition, … and output, e.g., display and audio, and other particular specifications of the device, e.g., display resolution, color capability, ….
- User: service selection preferences, content preferences and specific information about the user.
- Network connection: bandwidth, delay, and bit error rate.

Authors also present several requirements concerning the representation of context information. Accordingly, they defend that a context profile should be:

- Structured, to ease the management of the amount of gathered information and remark relevant data about the context.
- Interchangeable, for components to interchange context profiles.
- Composable/decomposable, to maintain context profiles in a distributed way.
- Uniform, to ease the interpretation of the information.
- Extensible, for supporting future new attributes.
- Standardized, for context profile exchanges between different entities in the system.

##### 2004, Gu *et al.*: The SOCAM Ontology

In 2004 Gu *et al.* [51] design the SOCAM service-oriented context-aware middleware architecture for designing and prototyping applications in an Intelligent Environment. following components:

- Context providers, which abstract context information from heterogeneous sources and semantically annotate it according to the defined ontology.
- The context interpreter, which provides logic reasoning to process information about the context.
- The context database, which stores current and past context ontologies.
- Context-aware applications, which adapt their behavior according to the current context situation.
- The service-locating service, which allows context providers and interpreters to advertise their presence for users and applications to locate them.

Gu *et al.* use OWL to describe their context ontologies in order to support several tasks in SOCAM. As the pervasive computing domain can be divided into smaller sub-domains, the authors also divided the designed ontology into two categories:

- An upper ontology, which captures high-level and general context knowledge about the physical environment.
- A low-level ontology, which is related to each sub-domain and can be plugged and unplugged from the upper ontology when the context changes.

As a result, the upper ontology considers person, location, computational entity and activity as context concepts.

Gu *et al.* [52] also present an OWL based model to represent, manipulate and access context information in smart environments. The model represents contexts and their classification, dependency and quality of information using OWL to support semantic interoperability, contextual information sharing, and context reasoning. The ontology allows to associate entities' properties with quality restrictions that indicate the contextual information quality.

##### 2005, Chen *et al.*: The CoBrA Ontology

Another work under a similar approach is the one performed by Chen *et al.* [53]. Authors introduce the CoBrA ontology based system, which provides a set of semantic concepts for characterizing entities such as people, places or other objects within any context. The system provides support for context-aware platforms in runtime, specifically for Intelligent Meeting Rooms. The context broker is the central element of the architecture. This broker maintains and manages a shared context model between agents (applications, services, web services, *etc.*) within the community. In intelligent environments participating agents often have limited resources and capabilities for managing, reasoning and sharing context. The broker's role is to help these agents to reason about the context and share its knowledge. The presented ontology relies on:

- Concepts that define physical places and their spatial connections.
- Concepts that define agents (humans and not humans).
- Concepts that define the location of an agent.
- Concepts that describe an agent activity.

##### 2005, Yamabe *et al.*: The Citron Framework

In 2005 Yamabe *et al.* [54] present a framework for personal devices which gathers context information about the user and his/her surrounding environment. Muffin, a personal sensor-equipped device is designed. Using it, several context parameters are gathered.

Sensor information is also considered for evaluating several user high-level context information. For example, accelerometer readings might recognize a walking or running activity, shaking and rotating, *etc*. The microphone is not only used to measure the ambient noise. It is also useful for detecting the place where the user is, e.g., meeting room, restaurant, street, ….

The context acquisition is categorized in two different groups: the user and the environment. For the user several issues are analyzed. For example, activities recognition requires the user to use the device in specific ways (it is not the same to use it with hands or waist-mounted). Another problem they encounter is about the time consuming process since an event is captured, then processed and finally validated. The last contextual issue deals with the intrinsic complexity and ambiguity of context information. For example, the meaning of what is loud might depend on the current situation, e.g., if the user is in a meeting room, if the user is in the street…. For the environment, Muffin suffer several heat problems due to the sensors sensitivity to environment temperature. This way, sometimes the gathered measures are invalid.

##### 2008, Wood *et al.*: AlarmNet

AlarmNet is an AAL monitoring wireless sensor network system (WSN) for pervasive adaptive healthcare in assisted living residences for patients with special needs. In this work, Wood *et al.* [55] contribute with several novelties:

- An extensible heterogeneous network middleware.
- Novel context-aware protocols.
- SenQ, a query protocol for efficiently streaming sensor data.

The context-aware protocol uses a two-way network information flow. On the one hand, environmental, system and residents data flow. On the other hand, circadian activity rhythm (CAR) analysis goes back into the system to enable smart power management and dynamic alerts.

Several sensors are used for sensing environmental quality: light, temperature, dust, resident activities (motion sensors), …. The devices' queries to the system are negotiated by the Query Manager.

##### 2011, Baltrunas *et al.*: InCarMusic

Assuming that context-aware systems adapt to the specific user situation, Baltrunas *et al.* present a music recommendation system which takes into account the user's mood and the traffic conditions [4]. To this end, the authors design a methodology where users can be requested to judge several contextual factors, e.g., if current traffic conditions are relevant for a decision making task, and to rate an item when a certain contextual condition is met.

In order to take into account the user's music preference and the influence that context might have into them, context is modeled as several independent factors.

##### 2012, McAvoy *et al.*

In this work the authors propose an ontology-based system for the managing of context within smart environments. One of the most significant contributions deals with the high-level information managed through the metadata and meaning which are collected by the sensor network [56]. The sensing devices within the smart environment have to be modeled in order to be semantically enriched. Data is formally represented as an ontology by using entities and the relationships which link them together. After the data is collected from the sensors it is passed to an enrichment module where it is made semantically rich. This new enriched data is stored in a semantic repository in the form of triples. The meaning of this information and the metadata are added to the data within this enrichment component.

##### 2013, Almeida and López-de-Ipiña: The AMBI2ONT Ontology

Almeida and López-de-Ipiña [57] consider two common problems dealing with ambiguity in the area of context modeling: the uncertainty and the vagueness. The uncertainty models the likeness of certain fact, while the vagueness represents the degree of membership to a fuzzy set. The uncertainty is represented by a certainty factor.

Due to the nature of the process of collecting data from the environment, the proposed ontology has been designed to support two types of uncertainty:

- Uncertain data: This kind of uncertainty is generated from the capture of data from sensors due to the imperfect nature of the devices.
- Uncertain rules: It occurs in the execution of the rules.

To reason over the ambiguous information the JFuzzy Logic Open Source fuzzy reasoner has been adapted to support uncertainty information.

#### 2.2.2. Context Models Comparison

The previous section has reviewed several significant context-aware systems and the followed approaches for modeling relevant context parameters of the environment depending on the application domain. In fact, this is one of the main problems in context modeling: the lack of model independence from similar domains and, also, the lack of models to be compared. Despite the fact that sometimes the “primary” domains are similar (context-aware computing, pervasive environments and ubiquitous computing) regarding the necessity of managing context knowledge, the concrete applications and approaches domains are different. Here, Henricksen *et al.* [49] realize about the lack of formality and expressiveness of previous context models.

However, to avoid this problem Gu *et al.* [52] present an ontology-based solution in which context information is modelled in two separated layers:

- In the upper layer there is an ontology which describes high-level knowledge about the current context and physical environment.
- Under it there is the possibility to add and remove ontologies which model low-level information of the current specific domain.

On the one hand, this approach allows developers to consider richer information, as activities, and abstract knowledge about the current global context. On the other hand, it makes possible to model specific knowledge of the current sub-domain. Besides, the possibility to plug and unplug these low-level ontologies makes this solution powerful. The solution provided by Gu *et al.* [51] is significant because of the following reasons:

- It considers high-level information on top of a more specific and domain dependent sub-model.
- Activities are modeled as a relevant concept for context in the upper ontology.
- The modeled entities are related. Persons are associated with locations and activities, each location is linked with indoor or outdoor entities, activities can be categorized into scheduled or deduced ones, *etc*.

As the work by Chen and Kotz [47] is a survey of context-aware computing the remarked context characteristics (location and time) are given more as an advice, since there is no model in their work. Nevertheless, they introduce several novelties like the term “context history”, which might be useful for future predictions about user's behavior and trends.

Modeling high-level information allows to perform deeper computations taking into account behavioral characteristics, trends information, inferred knowledge from small pieces of information combinations, *etc*. As can be seen in Table 2 many authors work with high-level data in their context-aware systems.

On the other hand, physical context parameters are frequently modeled in the analyzed literature. Location, time and environment conditions, e.g., temperature, pressure, light, noise, … are usually modeled to achieve final system's goal, e.g., adapting the user interface, recommending items or services, filtering resources, …. Besides, several approaches take user related characteristics to fulfill their purposes. For example, Schmidt *et al.* [43] consider not only physical environment as context information but also several human factors categorized as follows:

- User information, which gathers knowledge about user's habits, emotional stated and bio-physiological conditions.
- Social environment, made up by others users location, social interactions of the current user and knowledge about the behavior of groups of people.
- Task, taking into account spontaneous activities, engaged tasks and general goals.

A user modeling approach which considers emotional issues of the user is presented by Pereira [26]. As is remarked in Section 2.1.1. the author distinguishes between emotions and perceptions to separate both concepts for modeling processes. These approaches are found useful for recommending systems in which user mood and psychological state are relevant to filter multimedia content [4]. Following a similar perspective Evers *et al.* [34] analyze the user's participation in adaptive applications. Several concepts that should be taken into account regarding the user behavior and interaction with the application are presented (see Section 2.1.1.). Another similar approach regarding the user's more psychological aspects is presented by Liao *et al.* [35] Here the authors present a model for modeling user stress levels. This is related to the concept of Considerate Computing, term that it is explained in [58].

Schmidt *et al.* [43] also remark social environment as relevant for context modeling. More related to recommending environments, non explicit information about user's likes need to be computed. Similar approaches in these environments tend to avoid recommending systems intrinsic problems, like the so-called “cold start problem” [59,60].

Another relevant point remarked by Schmidt *et al.* [43] are the user's tasks. Several authors consider activities relevant for modeling context [49,51,61]. Activities enrich context information about the user [21]. It is common to model user activities in the user model (see Table 1).

Finally, and as occurs with user information, sometimes the collected data might lead to misunderstandings or non-trustworthy data. Almeida and López-de-Ipiña [57] consider ambiguity and uncertainty in their work and presents an ontology-based process which allows to model them within a smart environment.

Table 2 shows the differences between each reviewed solution for modeling context. It is difficult to gather all the approaches in a unique table. Therefore, Table 2 just emphasizes several differences, although these solutions have more characteristics modeled. For example, the work by Almeida and López-de-Ipiña [57] is focused on modeling context uncertainty and vagueness due to the nature of context data from sensors. Besides and as it is deeply discussed in [40], modelling the context with ontologies offers the following advantages:

- Ontologies are the most expressive approach to model context.
- Composition and management of the model can be done in a distributed manner.
- It is possible to partially validate the contextual knowledge.
- One of the main strengths of ontologies is the simplicity to enact the normalization and formality of the model.

Regarding the context-aware systems, we cannot go further without remarking the issues of gathering information from different sources or sensors. This information might sometimes be unreliable. Sensors can fail in the collecting process, they also can stop working due to several reasons, e.g., power or malfunction. What is more, every sensor “speaks” its language (this issue is addressed applying different techniques, like data fusion). Therefore, a process to evaluate the quality and trustworthiness of the collected information should be included in every context-based system as a method to avoid undesired results.

### 2.3. Device Models

There are many and different approaches in the literature for devices modeling. Devices capabilities might determine the boundaries of an adaptation process or the consumable resources. Lemlouma and Layaïda is considered that an independent device model would probably reduce adaptation process efforts for different context situations [62]. In fact, the World Wide Web Consortium (W3C) reinforces this perspective by warning about the wide range of device capabilities and sizes [63] which define the boundaries of the content that each device can handle. Several techniques, like Device Descriptors, content transformation guides, devices APIs and CC/PP systems help developers to optimize the user experience. These modeling techniques are analysed in the following section.

#### 2.3.1. Composite Capabilities/Preference Profiles

Composite Capabilities/Preference Profiles [64] (CC/PP) is a W3C standard system to express user and device capabilities. Using CC/PP a user will be able to show a specific preference or disability. For example, even though a user's device can display millions of colors, perhaps the user can just distinguish between a small set of colors. The necessity of this system stems from the wide range of web and ubiquitous devices available in the market. These devices have more and more multimedia and Web capabilities. This makes troublesome to Web content providers to service their content to these devices keeping usability and user satisfaction [62].

However, managing a large number of devices is not a new problem. Many approaches have been proposed in the literature to tackle this situation. Most of them are based on content management. This approach considers different presentation alternatives. Depending on the client device, a presentation configuration is served. This way, in the content serving process there are two options: (a) the server chooses which is the best configuration for the device, and (b) the client decides what to do with the content. This approach is very easy to be performed since every device identifies itself against the server.

CC/PP is based on profiles. A profile contains components and attributes. Each component has, at least, one attribute, and each profile has at least one component. The main components are: the hardware platform, the software platform and single applications, e.g., a browser. Attributes can contain one or many values. For example, in case of the “hardware platform” component we can find the attributes *displayWidth* and *displayHeight*. These attributes have a single value. CC/PP uses RDF (Resource Description Language) as formal language to build these profiles. Table 3 shows several advantages and drawbacks of this approach.

#### 2.3.2. UAProf

User Agent Profile [65] (UAProf) is concerned with collecting wireless devices capabilities and preferences. This information is provided to content servers to easy the content format selection process.

UAProf is directly related to the W3C CC/PP specification and it is also based on RDF. This way, the document schema is extensible [66,67]. These files, usually served as application/xml mimetype, describe several mobile devices capabilities, e.g., vendor, model, screen size, multimedia capabilities, *etc*. Most recent versions have also information about MMS, video streaming and more multimedia features. UAProf profiles are voluntarily built by the vendors, e.g., Nokia, Samsung, LG, … for GSM devices, or by several telecommunications company for CDMA/BREW devices.

The system works as follows:

1. The device sends a header containing a URL and its UAProf within an HTTP request.
2. The server side analyses the received UAProf to adapt the content to the device's display size.
3. Finally, the server takes the decision and serves the corresponding items to the device.

However, this approach has several drawbacks:

- Not every device have a UAProf.
- Not every UAProf are available.
- UAProf data can contain schema or data errors.
- There is no industry-wide data quality standard for the data within each field in an UAProf.

#### 2.3.3. Device Description Repository

The Device Description Repository (DDR) is a concept proposed by the Device Description Working Wroup (DDWG), an organization within the W3C. The DDR is supported by a standard interface and an initial vocabulary core about devices' properties. Web content authors will use these repositories to adapt their content to these devices. This way, the Web content interaction with different devices will be easier. Screen size, input mechanisms, supported colour sets, known limitations, special features, *etc.* are stored in these repositories.

Here we present three of the most popular DDR systems:

##### WURFL

The Wireless Universal Resource FiLe (WURFL) is an XML based open source database which contains the characteristics and capabilities of a wide range of devices. These capabilities are classified into several groups. These groups are just a simple way to understand WURFL and its data. Its API is very easy to use and it has the advantage of providing a hierarchy able to *infer* several capabilities for devices which are still not present in the file.

The following are several capabilities used by Almeida *et al.* [68] for Imhotep, a framework which aims to ease the development of accessible and adaptable user interfaces:

- display: It contains information about the device screen, as the resolution, number of columns, orientation, *etc*.
- image_format: Supported image formats.
- playback: Supported video codec.
- streaming: Available streaming capabilities.
- sound_format: Supported audio formats.

WURFL has become the de-facto standard for mobile capabilities. Nevertheless, there are several free and open source alternatives that are growing within the community very fast.

##### OpenDDR

One of the previously cited open alternatives is OpenDDR [69]. This DDR also provides an API to access DDRs about devices capabilities. It has two main advantages:

- The conviction that the application will work with any W3C DDR API implementation.
- Adopting the W3C standard the Copyright of the interfaces is protected by the W3C against any intellectual property and patent claims.

Nevertheless, the OpenDDR API is complex. It does not provide an architecture approach like WURFL. This way, it assumes default values for unknown parameters, e.g., displayWidth = 800 pixels.

##### 51Degrees.mobi

It works similar to OpenDDR, with the difference that 51Degrees.mobi [70]. has two versions. The first one (Lite) is free to use and it gathers several devices capabilities. The second one (Premium) is not free, but it has more data about devices and automatic updates for the stored information.

#### 2.3.4. DDR Solution Comparison

The main problem with these solutions is their inability to provide all the information developers usually need. Many fields are empty (which means that default values are used) or with error data. Another disadvantage is that, for the interface adaptation domain, sometimes we may need dynamic information about the device. For example, the battery levels, or the available memory, can be crucial pieces of information before making any adaptation process. Table 4 depicts each solution's drawbacks and advantages.

#### 2.3.5. Ontologies

There are other alternatives for modeling devices. As occurs with users and context ontologies allow us to give some meaning to the modeled concepts and the collected information. From the reviewed ontologies we remark the SOUPA ontology which, in addition to consider context information, it models many different aspects of static mobile capabilities and characteristics [72]. Another significant ontology is the one presented by Hervás, Bravo and Fontecha [73] as part of the PIVOn ontology. The Device Model Ontology describes not only the device's capabilities but its relationships with the service and user ontologies, dependencies and other features.

#### 2.3.6. Device Discussion

Apart from all the cited techniques for considering devices and their capabilities, we need to remark how technology evolves in a way that makes difficult to predict how we will work with smart devices in the near future. A few years ago context was understood as the result of the mixture of every physical sensor deployed in the environment. Temperature, light, noise, … all these context variables have been historically collected through several sensors located strategically. Now these sensors (and more) are also embedded in these smart devices. They have become wearable, and using their capabilities of the devices they area embedded in they have improved their performance. Touchable screens, network capabilities, parallel computing resources and high storage space embedded in small devices have changed the way context and smart devices interact. This situation makes us contemplate how this interaction channel would be in the near future. Smart watches, glasses and other wearable devices are, undoubtedly, changing the way smart environments will sense, behave and react.

### 2.4. Related Standardization Work

Regarding the standardization, there are several initiatives (dealing with HCI) that, considering users and devices, are now presented.

#### The Web Accessibility Initiative

The Web Accessibility Initiative (WAI) [74] is focused on enabling people with disabilities to equally participate in the Web, e.g., including social inclusion, regarding not only their disabilities but the location and available infrastructure. On the other hand, there are also different approaches to standardize the content [75] and the presentation of the user interface,*i.e.*, the Pennsylvania State University [76] studies how to deal with W3C's standards, accessibility and usability issues. Besides, several workshops are proposed to keep investigating under this issue (see [77]).

#### The Uncertainty Reasoning for the World Wide Web Incubator Group

Considering the uncertainty of the collected context information, the W3C's Uncertainty Reasoning for the World Wide Web Incubator Group (URW3-XG) [78] aims to define the challenge of reasoning with and representing uncertain information through related WWW technologies. In 2008, the W3C Incubator Group released a report where there are many recommendations through different analysis to identify and describe not only potential uncertain situations but applicable methodologies and the fundamentals of a standardized representation to effectively use them.

Table 5 shows several W3C standards and different specifications considering users and devices. Besides, there are also many guidelines and discussion groups working on these issues.

The W3C remarks the lack of definition that makes the interoperability of user models difficult. In this context, they propose the following areas to work on:

- A standard interoperability model providing API's for different purposes and applications
- Common data storage format for user profiles
- Common calibration/validation technique
- Collaboration on ethical issues
- Ensuring sustainability by making them available within a standard
- Mechanisms for exchanging user profile data between sources
- Protection mechanisms for privacy issues
- Control mechanisms for user profile data exposure

##### The Video in the Web Activity

Promoting the use of the video in the Web, the Video in the Web Activity [79] aims to build a solid architecture to enable users to create, navigate, search, link and distribute video, effectively making video part of the Web. This activity group is formed by three different working groups:

- The Timed Text Working Group [80], which mission is to provide a language that represents textual information that is associated with timing information. This group has released two versions of the Timed Text Markup Language (TTML). The first one was released in 2010. The last one during 2013.
- The Media Annotations Working Group [81], which main purpose is to provide an ontology and API to facilitate cross-community data integration of multimedia information in the Web. The group has published the following documents:
  – A second version of Use Cases and Requirements for Ontology and API for Media Resource 1.0 on January 2010, as an input for the development of “the Ontology and the API for Media Resource 1.0” Specification.
  – A W3C Recommendation version of the Ontology for Media Resource on February 2012.
  – The group is now about to publish a Proposed Recommendation of the API for Media Resources.
- The Media Fragments Working Group [82], which successfully aimed to address temporal and spatial media fragments in the Web using URIs. The group was closed on December 2013 having successfully published two versions (basic and advanced) of the Media Fragments URI 1.0 specification as a Recommendation.

The Video in the Web Activity members are still working over the issue of the video codec to be used in the W3C specifications (in particular HTML5).

##### The Provenance Working Group

Provenance is defined as the information about the involved entities in producing data which can be used to form assessments about its trustworthiness. In order to allow users to mark up web pages using the terms provided or by making available provenance information expressed as linked data the PROV specification [83] provides a vocabulary to interchange this provenance information. This specification consists of 11 documents that define various necessary aspects to achieve the vision of inter-operable interchange of provenance information in heterogeneous environments such as the Web (we do not consider the Overview document). Table 6 shows the cited documents and their details. After the publication of the PROV Ontology [84] the PROV group was closed on June, 2013.

##### The Moving Picture Experts Group

The Moving Picture Experts Group (MPEG) is a working group of ISO/IEC with the mission to develop standards, e.g., MPEG-ACC, MPEG-H, MPEG-DASH, MPEG-4, … for coded representation of digital audio and video. The group has produced several standards that help the industry offer end users an ever more enjoyable digital media experience. As this group keeps working on the cited objectives the 108 meeting is scheduled March 2014 in Valencia, Spain [85].

##### The Internet Engineering Task Force

The mission of the Internet Engineering Task Force (IETF) is to produce high quality and relevant technical documents that influence people to design, use, and manage the Internet. IETF's standards development work is organized into 8 areas. Within each area there are multiple Working Groups. Each Working Group has one or more chairs who manage the work, and a written charter defining what the work is and when it is due. Table 7 shows the current active IETF Working Groups. The next meeting is scheduled for March, 2014 in London, England.

## 3. Discussion

Designing interactive systems entails several problems. First, the corresponding entities have to be selected. Next, we need to identify the set of capabilities and characteristics that would define each entity taking the current domain into account. These capabilities will characterize them not only in the model but within the whole environment. Third, we need to choose the technical approach to develop these models, *i.e.*, taxonomies, ontological, object oriented, *etc*. And finally, we have to develop such a system. Considering the reviewed literature we have identified several setbacks beyond the presented problems: dynamism, ignorance about user capabilities, uncertainty about collected information and domain dependency.

Modeling with dynamism requires several requisites. First of all, a powerful sensor environment is needed. The system performance should be good enough to maintain the information validity and coherence. This is because temporal constraints, which determine how useful a piece of information is in the current context. Furthermore, information must be heterogeneous. Each entity should be independent, but it also will need to understand others. One of the main problems found in this review is that usually user's characteristics are considered as static. In Section 1.1 we introduce the concept of “context disabilities” which already address this point. Following this perspective we remark here the works by Gregor *et al.* [5], Razmerita *et al.* [21] and Skillen *et al.* [36] in which user models are maintained and updated.

Regarding the users, we deem that it is hard to model and understand their capabilities. Several approaches use capabilities information to perform adapted interaction with the user. Nonetheless, we think this approach is not very accurate. It would be desirable to have some physiological background to be able to model each capability of each user. Nevertheless, the work by Casas *et al.* [32] makes this possible by making an abstraction about the capabilities and regarding more the user needs.

Trusting the collected data from from users and context is also a significant problem. Usually context data come from sensor networks. Sensors can stop working or worse, which would be if they start giving non-sense data. Razmerita *et al.* [21] avoid this issue by collecting explicit data from the user. On the other hand, Almeida and López-de-Ipiña [57] deal with this problem by using an ontology and a reasoning engine which are capable of working with ambiguity and uncertain data.

Finally, another problem that we emphasize in this paper is the domain dependency of the analyzed solutions. This dependency makes difficult to reuse and export these approaches in/to different domains. This is clearly shown in the presented Tables 1 and 2. Looking at the considered characteristics (the columns) we can see how different solutions deal with similar aspects. The problem comes when other solutions are not taken into account. Besides, modeling with ontologies have the advantage of reusing these ontologies, parts or classes that have been designed by others.

Regarding at the modeling technique, we have remarked that Strang and Linnhoff-Popien [40] demonstrate that using ontologies might be more appropriate for modeling context. This technique is similarly useful for users and devices. In fact, several ontologies model these and more entities: FOAF [97], UserModelOntology [98], CoBrA/SOUPA [99], …. But, of course, it is not the only or the best solution for modeling these entities. This depends on the system characteristics and on the designer. Here we remark on several alternatives:

### Key-value

Maas *et al.* [100] adopted a X.500 based solution to store location data. This approach is also used in distributed searching systems. Although it is very easy to maintain and handle its main problem is that it makes difficult to build complex structures [40]. Similar solutions follow this key-value approach to identify a context element (key), like location, with an environment variable (value) [101].

### Markup scheme

Based on several derivative Generic Standard Markup Language (SGML), for example XML, marking schemes based models are widespread for modeling profiles. Some extensions are defined as Composite Capabilities/Preferences Profile (CC/PP) [64] standards and User Agent Profile (UAProf) [65], which have an achievable expressiveness by RDF and XML serialization. This kind of context modeling usually extends and completes the CC/PP and UAProf basic vocabularies. In [50] authors present an extension of this model, Comprehensive Structured Context Profiles (CSCP), which provides hierarchy to such schemes supporting the RDF flexibility to express natural structures of profile information.

### Graphic models

While Bauer *et al.* [102] used a UML tool to model the context in a air traffic domain, Henricksen *et al.* [103] presented a graphic model (as an extension of Object-Role Modeling [104]). UML (Unified Modeling Language) is a widespread general purpose modeling tool with a very powerful graphic component (graphic models): the UML diagram.

### Object oriented models

Strang and Linnhoff-Popien [40] presented an object oriented model in which context process details are embedded into object level. Data is hidden from other components. This way the access to this context data is just allowed through several interfaces. This approach tries to use the object oriented programming benefits, as re-usability and encapsulation, to cover ubiquitous environment's problems about context. Another example of this approach is the one given by the GUIDE project by Cheverts *et al.* [105], which is focused on location. In this case the context information is also in the object as accessible states through those methods defined by the object itself and by modifying these states.

Users might experience several issues when interacting with user-oriented systems and unknown interfaces. Usability and acceptance need to be considered in this area. Ziefle, Holzinger and Röcker [106] study how users between 17–95 years accept smart technology at home as familiar. They demonstrate that users sociable people (considering their social contacts) and a high interest in technology show higher acceptance for these smart services at home. Besides, in [107] the authors address the problematic of elder users handling modern interfaces within AAL environments. Elderly users are trained in a laboratory where they interact with different interfaces. The authors extract several conclusions about the usability, acceptance and suitability of the presented interfaces. Moreover, Holzinger *et al.* [11,108] also discuss about the necessary trends to include the elderly making technology more accessible, usable, and enjoyable.

## 4. Conclusions

In this paper, we have reviewed the current state-of-the-art in modeling users, context and devices from the HCI perspective considering AAL and Pervasive Computing environments. On the one hand, user modeling aims to benefit users in an interaction domain. On the other hand, modeling context is challenging due to the necessity of a sensor infrastructure. Finally, several techniques for modeling devices and their capabilities are introduced.

Hopefully the discussions along this paper (see Section 2.1.2., Section 2.2.2. and, finally, Section 3) will make future researchers consider several advantages and drawbacks when they have to design different models.

Finally, the main objective of this paper is not only to make a review of the state-of-the-art. We have tried to point out several problems that future researchers might face. Modeling agents within a smart, HCI or pervasive environment will make them researchers to face similar problems. This means that, despite these domains are different, the processes to get to their goals will be similar. Besides, it is necessary to remind that open and modular solutions (like the analyzed ontology-based approaches) help developers to reuse, understand and extend others' work, which we think enriches the scientific community in this area. Moreover, we are developing a dynamic and adaptive user interface system for mobile devices considering these approaches. We have chosen ontologies as the modeled technique for characterizing users, context and devices, and the design process is ruled by these literature approaches. In other words, some of the reviewed solutions are being used and extended although the main domain deals with adaptivity.

## Figures and Tables

**Table 1.** Related work for the user modeling approaches. Under the user characteristics heading Activities or behavior, Capabilities, Experience, Interests, Emotions, Personal, Stress and Location information are presented.

**Solution**	**Domain**	**Ontologies**	**User characteristics**							

			**A**	**C**	**Ex**	**I**	**E**	**P**	**S**	**L**
2002, Gregor *et al.* [5]	Inclusive design			✓	✓					
2003, Gauch *et al.* [20]	Automatic profiling	✓				✓				
2003, Razmerita *et al.* [21]	KMS	✓	✓			✓		✓		
2005, Hatala and Wakkary [22]	Tangible interfaces	✓				✓		✓		✓
2005, Fernando Pereira [26]	Multimedia adaptation						✓			
2007, Persad *et al.* [27]	Product design demands		✓	✓						
2007, Golemati *et al.* [29]	User profiling	✓	✓		✓	✓		✓		
2007, Heckmann *et al.* [30]	Ubiquitous applications	✓	✓				✓	✓	✓	
2008, Casas *et al.* [32]	Adaptive user interfaces			✓	✓					
2012, Evers *et al.* [34]	Adaptive applications								✓	
2012, Skillen *et al.* [36]	Mobile environments	✓	✓	✓		✓				✓

**Table 2.** Related work for context modeling approaches. Under the domain heading Context Awareness, Context Modeling, Pervasive Computing, Smart Environments, Mobile Computing and Recommender Systems. Under the context parameters heading Location, Time, Activity, Nearby Resources, Nearby People, Physical Environment, Social environment, Infrastructure, User's parameters and High level information are presented.

	**Domain**	**Ontologies**	**Context parameters**									

			**L**	**T**	**A**	**R**	**P**	**E**	**S**	**I**	**U**	**H**
2000, Chen and Kotz [47]	CA		✓	✓								
2001, Anthony Jameson [44]	CM		✓		✓						✓	✓
2002, Henricksen *et al.* [49]	PC				✓	✓			✓	✓		✓
2002, Held *et al.* [50]	CA									✓	✓	
2004, Gu *et al.* [51]	SE	✓										✓
2005, Chen *et al.* [53]	PC	✓	✓	✓	✓	✓	✓	✓		✓	✓	
2005, Yamabe *et al.* [54]	MC		✓		✓			✓			✓	✓
2008, Wood *et al.* [55]	AAL				✓			✓			✓	
2011 Baltrunas *et al.* [4]	RS							✓			✓	
2012, McAvoy *et al.* [56]	SE	✓					✓					✓
2013, Almeida and López-de-Ipiña [57]	SE	✓	✓					✓				

**Table 3.** CC/PP: several advantages and drawbacks.

**Advantages**	**Drawbacks**
A good infrastructure for modeling devices	Device dependent
Content negotiation flexibility	It requires a more mature user preferences definition
Using CC/PP, Web based device developers and user agents can define accurate profiles for their products. Web servers and server proxies can use these profiles to perform the adaptation	
Open to new protocol proposals for profile exchanging	

**Table 4.** Analysed DDRs comparison [71].

**DDR**	**Advantages**	**Drawbacks**
WURFL	Upgradeable to new versions	Errors in data
	A hierarchy which allows to infer values	Many empty values
	Many capabilities modelled	
	Very easy to configure	
	Powerful API	

OpenDDR	Free to use, even commercially	Limited number of capabilities
	Growing community	Default values for unknown data

51Degrees.mobi	A Lite version, free to use even commercially	Limited number of capabilities
	Easy to install and use	

**Table 5.** W3C standards and specifications for user and device modeling.

**Entity**	**Contribution**	**Type**	

		**Standard**	**Specification**
User	UAProf	✓	
	Multimodal Architecture and Interfaces	✓	
	Extensible MultiModal Annotation markup language	✓	
	Ink Markup Language (InkML)	✓	
	Accessibility (All)		✓
	Web Content Accessibility Guidelines (WCAG)		✓
	Accessible Rich Internet Applications (WAI-ARIA)		✓
	User Agent Accessibility Guidelines (UAAG)		✓
	Authoring Tool Accessibility Guidelines (ATAG)		✓
	Evaluation and Report Language (EARL)		✓
	IndieUI		✓
Device	CC/PP	✓	
	Introduction to Model-Based User Interfaces		✓
	Model-Based User Interfaces Glossary		✓
	Guidelines for writing device independent tests		✓
	Delivery Context Overview for Device Independence		✓
	Authoring Techniques for Device Independence		✓
	Device Independence Principles		✓
	Authoring Challenges for Device Independence		✓
	Delivery Context Ontology		✓
	Delivery Context: Client Interfaces (DCCI)		✓

**Table 6.** PROV documents description. Document type (Recommendation or Note) is also shown.

**Document**	**Type**	**Details**
PROV-PRIMER [86]	Note	The entry point to PROV offering an introduction to the provenance data model
PROV-XML [87]	Note	Defines an XML schema for the provenance data model PROV data model
PROV-O [88]	Rec.	PROV-O defines a light-weight OWL2 ontology for the provenance data model
PROV-DM [89]	Rec.	Defines a conceptual data model for provenance including UML diagrams. PROV-O, PROV-XML and PROV-N are serializations of this conceptual model
PROV-N [90]	Rec.	Defines a human-readable notation for the provenance model.
PROV-CONSTRAINTS [91]	Rec.	Defines a set of constraints on the PROV data model that specifies a notion of valid provenance
PROV-AQ [92]	Note	Defines how to use Web-based mechanisms to locate and retrieve provenance information
PROV-DC [93]	Note	Defines a mapping between Dublin Core and PROV-O
PROV-DICTIONARY [94]	Note	Defines constructs for expressing the provenance of dictionary style data structures
PROV-SEM [95]	Note	Defines a declarative specification in terms of first-order logic of the PROV data model
PROV-LINKS [96]	Note	Defines extensions to PROV to enable linking provenance information across bundles of provenance descriptions

**Table 7.** Active IETF Working Groups.

**Working Group**
Applications Area Working Group
Constrained RESTful Environments
Extensible Provisioning Protocol Extensions
Hypertext Transfer Protocol Bis
BiDirectional or Server-Initiated HTTP
JSON data formats for vCard and iCalendar
JavaScript Object Notation
Protocol to Access WS database
Preparation and Comparison of Internationalized Strings
IMAP QRESYNC Extension
System for Cross-domain Identity Management
SPF Update
Uniform Resource Names, Revised
Using TLS in Applications
Web Security
Web Extensible Internet Registration Data Service
